# Structural and Techno-Functional Properties of Bovine Collagen and Its Application in Hamburgers

**DOI:** 10.17113/ftb.57.03.19.5896

**Published:** 2019-09

**Authors:** Christian Alexandretti, Roberto Verlindo, Guilherme De Souza Hassemer, Alexandra Manzoli, Silvane Souza Roman, Ilizandra Aparecida Fernandes, Geciane Toniazzo Backes, Rogério Luis Cansian, Mônica Beatriz Alvarado Soares, Rodrigo Schwert, Eunice Valduga

**Affiliations:** 1 Department of Food Engineering, URI Erechim, Av. Sete de Setembro 1621, Erechim, RS, 99709910, Brazil; 2 Department of Chemical Engineering, Federal University of Santa Maria, Camobi, Santa Maria, 97105900, Brazil

**Keywords:** collagen, soybean flour, technological properties, gel strength, hamburger

## Abstract

The objective of this work is to characterize two types of bovine collagen (fibre and powder), evaluating its application in mixed hamburger formulations, as well as the quality characteristics of the products. The collagen fibre had a fibrillar structure, molecular mass 100 kDa and greater gel strength (146 315 Pa) and protein content (97.81%) than the powdered collagen, which had molecular mass from 50 to 100 kDa, greater hydroxyproline content, and a morphological structure with spherical microparticles more amorphous than the collagen fibre. In this study we found that the addition of 1.5% powdered collagen and 2.5% flocculated soybean flour and/or 0.75% powdered collagen and 3.5% flocculated soybean flour did not deteriorate the technological properties or the sensory attributes of hamburgers. The use of collagen is a promising alternative, since it has functional properties, improves the texture characteristics of a product, and is of low cost.

## INTRODUCTION

Meat products, such as frankfurters, salami, mortadella, sausages, crumbed products, meatballs and hamburgers, are attractive products for consumers as they require little preparation. Among the so-called fast food, hamburgers stand out as an excellent choice due to their sensorial characteristics, nutritional value, low price, and ease of preparation.

According to Brazilian legislation (*1*), hamburgers are meat products manufactured on an industrial scale from minced meat, with or without adipose tissue, with characteristic texture, colour, and flavour, moulded and submitted to appropriate technological processes. However, during hamburger preparation and cooking some problems, such as shrinking, mass loss and reduced yield, may arise. The use of alternative ingredients, such as non-meat proteins (isolated, concentrated, textured, and flocculated soy protein) at 4% maximum mass fraction has been used by industries in order to minimize the above-mentioned issues. Fibre or powdered collagen could also be used in hamburgers since they increase their nutritional value and help to reduce deformity and mass loss during thermal treatment. Furthermore, some of their advantages include cost reduction, higher protein content, as well as improved functional properties, such as increased capacities of water absorption, gel formation, stabilization and emulsion formation (*2*), at 1.5% maximum mass fraction in meat products. Thereby, collagen preparations can be used to improve processed meat attributes since, at low levels, functional collagen proteins stabilize shrinkage and promote increased cooking yield due to their gelling and water-binding properties (*3*–*5*).

Collagen is one of the most useful biomaterials due to its wide range of industrial applications (*6*). Bovine and chicken skins predominantly contain type I and III collagen fibrils (*7*). On a molecular basis, fibril-forming collagen features an uninterrupted helical region with alternating polar and non-polar domains leading to a lateral alignment of molecules in a quarter-staggered array (*8*). Type I collagen is a heterodimer composed of two identical α1-chains and one α2-chain (*7*), whereas type III collagen is a homotrimer, with three α1-(III)-chains and usually occurs in the same fibril with type I collagen (*9*). Collagen stability and structure are based on hydrogen bonds between polar residues of 4-hydroxyproline, 5-hydroxylysyl hydration networks and electrostatic interactions (*7*). The last emerge between ionizable side groups present in 15–20% of all amino acid residues, either in X or Y position of the Gly-X-Y triplets (*10*).

Due to collagen low production cost and functional properties, its use as an additive allows for a cheap alternative to improve meat product texture, resulting in an improved organoleptic bite sensation, without a significant increase of the product price. Thus, the aim of this study is to characterize two types of bovine collagen (fibre and powdered collagen), evaluating their application in mixed hamburger formulations, as well as the quality characteristics of meat products in an industrial unit. The results will provide information about the technological properties and chemical characteristics of bovine collagen (fibre and powdered) and the prospects of its industrial applications.

## MATERIALS AND METHODS

### Collagen characterization

Bovine collagen fibre (*d*_particle_=1.80–1.90 mm) and collagen powder (*d*_particle_=0.45–0.6 mm) were supplied by Novaprom Food Ingredients Ltda (Guaiçara, Brazil). Hydroxyproline and total protein content in the collagen samples were determined and protein fractions were identified by sodium dodecyl sulfate–polyacrylamide gel electrophoresis (SDS-PAGE), morphological structure using scanning electron microscope (SEM) technique, chemical composition with X-ray photoelectron spectroscopy (XPS), and crystallinity through X-ray diffraction (XRD).

### Total protein and hydroxyproline

The protein and hydroxyproline contents in collagen samples were quantified according to AOAC method 981.10 (*11*) and AOAC method 991.20 (*12*), respectively.

### Gel strength

Initially, fibre and powdered collagen gels were prepared at a 1:6 (*m*/*V*) ratio, heated and homogenized until reaching 72 °C. After that, the solution was cooled down to 8 °C for at least 12 h. Gel strength was then established using a TA.XT2 texture analyser (Stable Micro Systems Ltd, Godalming, Surrey, UK) with a 10-kg load cell, and pre-test, test, and post-test speeds of 1, 1 and 10 mm/s, respectively, as well as a 0.5-inch diameter spherical probe.

### SDS-PAGE electrophoresis

Sodium dodecyl sulfate-polyacrylamide gel electrophoresis (SDS-PAGE) was performed according to the method proposed by Laemmli (*13*) and Bustamante-Vargas *et al*. (*14*). First, the powdered and fibre collagen samples were prepared at a concentration of 5.0 and 2.5 mg/mL, respectively. For sample preparation, 40 µL of 60% trichloroacetic acid (*m*/*V*) (Labsynth, Diadema, Brazil) were added to 100 µL of raw samples, placed in Eppendorf tubes and stored overnight in a freezer at –15 °C. The samples were subsequently centrifuged (model 5403; Eppendorf, Hamburg, Germany) at 10 000×*g* and 4 °C for 30 min, and then the supernatant was removed.

### Scanning electron microscopy

Fibre and powder collagen morphology has been analysed by scanning electron microscopy (SEM microscope model JSM-6510; JEOL, Austin, TX, USA). The sample surfaces were coated with a gold layer (approx. 20 nm) using a sputter coater (model BAL-TEC SCD 050; BAL-TEC AG, Balzers, Liechtenstein).

### X-ray photoelectron spectroscopy

Surface chemical composition analysis was done using X-ray photoelectron spectroscopy (XPS model Escalab 250Xi; Thermo Fisher Scientific, Lafayette, CO, USA) attached to a scanning electron microscope (SEM model JSM-6510; JEOL). In addition, XPS mapping approach was used to study chemical element dispersion on the electrode surface.

### X-ray diffraction

Collagen physical structure (crystalline and/or amorphous) was characterised by X-ray diffraction (XRD) using a diffractometer (model XRD-6000; Shimadzu, San Diego, CA, USA) with CuKα radiation, from 10° to 80° (2*θ*) at 2°/min.

### Hamburger formulation preparation

Elaboration and characterization of the hamburgers was carried out entirely in a large industrial unit (Santa Catarina, Brazil). Mixed meat (chicken and pork) hamburger formulations were prepared according to the parameters set by the agribusiness industry, following legal standards (*1*). The formulations were made by varying the mass fractions of fibre and powdered collagen (0.75–1.50%) and flocculated soy protein (Grupo Bremil, Arroio do Meio, Rio Grande do Sul, Brazil) (2.5–4.0%): P (standard formulation)=4.0% flocculated soy protein, T1 formulation=3.25% flocculated soy protein and 0.75% collagen fibre, T2 formulation=2.50% flocculated soy protein and 1.50% collagen fibre, T3 formulation=3.25% flocculated soy protein and 0.75% powdered collagen, and T4 formulation=2.50% flocculated soy protein and 1.50% powdered collagen.

Only chicken and pork meat (undeclared quantities; industrial formulation) were used, using cuts with no obvious fat and with minimum visible connective tissue (pork loin and chicken breast). The fat used was removed from the pork loins and the cuts of meat were kept frozen (maximum 0 °C).

First, the meat was cut using a mini cutter (Incomaf Indústria Ltda., São Paulo, Brazil) for 30 s, and then mixed with other ingredients. Then, the meat with soy protein and collagen was homogenized, hydrated with water for 15 min, and subsequently mixed using a blender (Risco, São Paulo, Brazil) for 3 min. A second grounding using a meat grinder (Seydelmann, Stuttgart, Germany) with a 5-mm disc was performed in order to standardize particle size.

After formulation preparation and grinding, the hamburger mix was submitted to a moulding stage. The process was done using a manual moulding equipment producing 90 g patties, after which the samples were frozen at –9 °C for a period of 90 days.

### Hamburger characterization

Protein, moisture, fat, hydroxyproline, mass loss, instrumental texture (hardness), as well as histological and sensorial characteristics of the hamburger formulations were characterized on the first storage day.

#### Physical and chemical characteristics

Protein, hydroxyproline, moisture and fat contents were determined according to AOAC methods 981.10 (*11*), 991.20 (*12*), 985.26 (*15*) and 991.36 (*16*). Mass loss during cooking was assessed after heat treatment using the grill or oven. The grill (model ED 36G; Garland, Mississauga, Canada) was prepared by spraying with cooking oil and preheating for 2 min. Then, the frozen hamburgers were placed on the preheated grill and cooked for 3 min on each side. The oven (model Picasso; Venax®, São Paulo, Brazil) was preheated at 250 °C for 5 min, the frozen hamburgers were baked in the oven for 15 min (7.5 min on each side). The internal temperature of the product was kept at minimum 72 °C.

To determine mass loss, hamburgers were weighed on an analytical balance (model MA035; Marconi, Piracicaba, Brazil) before and after each heat treatment. Hardness was determined by sample compression method by a computer-controlled TA.XT2 texture analyzer (Stable Micro Systems Ltd.), with a Warner-Bratzler blade, equipped with a 10-kg load cell, using a 6.35-cm cylindrical probe. The pre-test, test, and post-test parameters were 2, 1 and 7 mm/s, respectively. Samples of the product ready for consumption of about 10 mm in height and compressed to 25% of their size were analyzed in accordance with Harper *et al.* (*17*).

#### Histological analysis

For the histological analysis, the hamburger samples from each formulation were fixed at 10% formalin and subjected to routine histological techniques, including gradual dehydration, diaphanization, infiltration steps, and embedding in paraffin. From each paraffin block, 4 μm thick histological sections were taken and the sections were stained with hematoxylin-eosin (*18*). The histological sections were analyzed using a microscope (model Lambda LQT-3; ATTO Instruments Co, Hong Kong, PR China) with the images photographed with a Motic Images Plus v. 2.0 software (Motic China Group Co. Ltd., Beijing, PR China) (*19*). The histological field of each slide was evaluated using 10× and 25× magnification.

#### Sensory evaluation

Sensory evaluation of the hamburgers was performed on a laboratory scale, with 12 trained panellists, who were employees of the meat processing industry, male and female, aged from 20 to 50. The sensory evaluation of hamburgers was conducted on the first day, serving 90-gramme samples grilled and baked in the oven (according to the procedure described in the section Physical and chemical characteristics).

The hamburger samples were coded with randomized three-digit numbers, and distributed along with the evaluation form and blank samples (cracker and mineral water). The panellists assessed each attribute (flavour, colour, odour, appearance, texture, and general acceptance) on a 9-point hedonic scale (1=dislike extremely and 9=like extremely), according to the procedure described by Queiroz and Treptow (*20*).

As the research involved humans, tests were performed according to the Research Ethics Committee of the Regional Integrated University of Upper Uruguay and Missions, as well as Brazilian National Health Council ethical and scientific requirements, registered at Plataforma Brasil (*21*).

### Statistical analysis

The results (*N*=3) were analysed by analysis of variance (ANOVA), followed by the Tukey’s test to compare the average differences, using the Statistica v. 5.0 software (*22*), with a 95% confidence level. In addition, the Pearson correlation analysis and principal component analysis (PCA) were performed using XLSTAT software (*23*).

## RESULTS AND DISCUSSION

### Hydroxyproline and protein contents, and gel strength of collagen preparations

Table 1 shows the characteristics of fibre and powdered collagen samples. It can be noted that there was a significant difference (p<0.05) in the hydroxyproline mass fraction between the samples, with powdered collagen having higher value (2.06 g/100 g) than collagen fibre. Collagen fibre had (p<0.05) a slightly higher protein content (97.81 g/100 g) than the powdered one (96.87 g/100 g).

**Table 1:** Hydroxyproline and protein contents and gel strength of the collagen samples

Sample	*w*(g/100 g)		Gel strength/Pa
Hydroxyproline	Protein
Collagen fibre	(1.96±0.01)^b^	(97.81±0.11)^a^	(146 315±883)^a^
Powdered collagen	(2.06±0.01)^a^	(96.87±0.09)^b^	(91 888±4119)^b^

Mean values with the same letter in superscript within a column are not significantly different at 5% (Student’s *t*-test), *N*=3

Variations in protein and hydroxyproline contents are due to the raw material, collagen extraction process and origin. According to Gómez-Guillén *et al.* (*24*), high temperatures cause protein solubilization and greater fragmentation of the collagen structure.

Gauza-Włodarczyk *et al*. (*25*) state that whatever their origin, collagen contains 19 amino acids; two of them, hydroxyproline and hydroxylysine, are practically absent from other proteins. The same authors verified that the hydroxyproline content in bovine Achilles tendon collagen (8.15 g per 100 g of protein) is 30% higher than the hydroxyproline content of collagen from fish skin and concluded that fish skin collagen is less stable than the bovine Achilles tendon.

The gel strength of collagen fibre was significantly different (p<0.05) from the powdered one with a higher value (146 315 Pa), demonstrating that fibre allows greater solvent entrapment than powdered collagen gels. According to Prestes (*26*), gel resistance (Bloom force) depends on concentration and molar mass, where a higher Bloom value is correlated to collagen molar mass, hence a high Bloom value (about 300 g) results in firmer gels. The obtained data show that powdered and fibre collagen are different products with distinctive characteristics, possibly due to the extraction process used.

### SDS-PAGE of collagen preparations

**Fig. 1:**
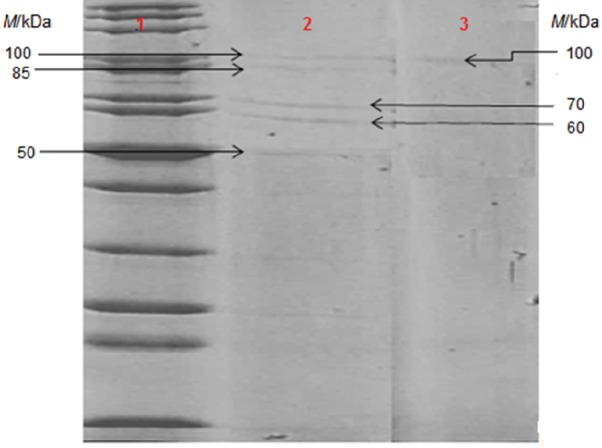
Sodium dodecyl sulfate-polyacrylamide gel electrophoresis (SDS-PAGE) for tested collagen. Legend: from left to right – column 1=molecular mass standards, column 2=collagen powder, column 3=collagen fibre

Fig. 1 shows SDS-PAGE for the tested collagen samples. On the electrophoresis gel, four distinct bands were identified for powdered collagen with molecular masses varying from 50 to 100 kDa, whereas a 100-kDa band for collagen fibre was found.

According to Oechsle *et al.* (*27*), SDS-PAGE gel with bovine telopeptide-poor collagen showed two distinct bands of approx. 123 kDa, indicating α1(I) and α2(I) chain monomers of type I collagen. Furthermore, the slightly larger band of α1(III) chain was observed above, indicating a type III collagen. The mass spectrometry analysis noted the presence of α1(I), α2(I) and α1(III) chains with 133, 129 and 138 kDa, respectively.

### Scanning electron microscopy of collagen preparations

**Fig. 2:**
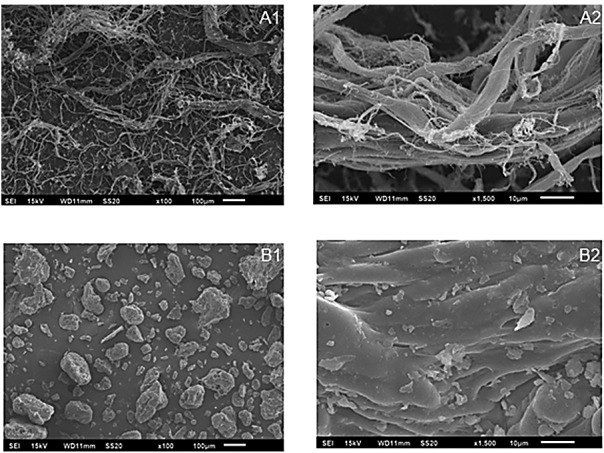
Micrographic images of collagen fibre (A1 and A2) and powder (B1 and B2). Magnification: 1=100× and 2=1500×

The SEM micrographs obtained for collagen fibre and powdered collagen are shown in Fig. 2. These results show that both materials had quite a different microstructure, with the powdered collagen (B1 and B2 in Fig. 2) having lost its fibrous characteristic during the milling of the collagen fibre (A1 and A2 in Fig. 2). Collagen fibre showed an internal axis with several thin ramifications connecting them to smaller external particles, whereas powdered collagen showed a major trend to form agglomerates. The thin filaments observed in the collagen fibre may be important in the eventual interaction between the polymeric matrix and fillers.

The greatest difference between fibre and powdered collagen is that the fibre physical structure retains water chemically, either through protein matrix or hydrogen bonding with water (*26*). As such, fibre swells in contact with water, blocking both moisture and fat from exiting the system.

### X-ray photoelectron spectroscopy of collagen preparations

The main elements found in both powdered and collagen fibre were carbon, nitrogen and oxygen (Fig. S1 and Fig. S2). Elemental mapping showed that such elements were homogeneously distributed on both powdered and collagen fibre surfaces. The presence of other elements, such as aluminium, magnesium, sodium and fluorine, was also noted in both collagen samples, while iron was found only in the powdered one. These differentiations could have been due to collagen production methods.

### X-ray diffraction patterns of collagen preparations

**Fig. 3:**
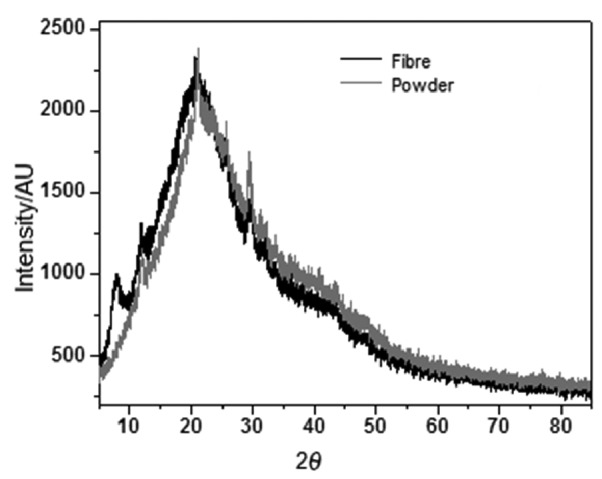
X-ray diffraction (XRD) patterns for collagen powder (grey line) and fibre (black line)

Fig. 3 shows great XRD pattern similarities of both fibre and powdered collagen samples. Collagen fibre preponderant peaks were obtained with 2*θ* at approx. 7°, 25° and 30°, and powdered collagen preponderant peaks were obtained with 2*θ* approx. 25° and 30°, in line with reported reticulated collagen values (*28*), typical for amorphous material. The results indicated that powdered collagen is more amorphous than the fibre.

### Physicochemical and sensorial characteristics of hamburger formulations

Average mass fractions and the respective standard deviations of protein, fat, moisture and hydroxyproline of different hamburger formulations on the first day of storage are shown in Table 2. The mass fraction of protein varied from 16.73 (T3) to 17.8% (T4). Formulations T1 and T3, with 0.75% fibre and powdered collagen respectively were not significantly different (p<0.05) from the standard one. Formulations T2 and T4, with 1.5% collagen fibre and powdered collagen respectively had higher protein content (p<0.05). The results for protein content suggest that the addition of 1.5% collagen (fibre or powdered) increased protein content. Such results are in accordance with Prestes *et al.* (*29*), who also reported total protein content increase in products containing bovine collagen.

**Table 2:** Chemical composition (protein, lipids, moisture and hydroxyproline mass fractions), hardness and mass loss (after heat treatment on the grill or in the oven) of the hamburger formulations on the first day of storage

Run	*w*(g/100 g)				Hardness/N	Mass loss/%	
Protein	Lipid	Moisture	Hydroxyproline	Oven Grill	
Standard	(16.93±0.08)^b^	(8.22±0.09)^d^	(67.6±0.1)^b^	(0.16±0.05)^d^	(59.7±5.2)^c^	(36.2±0.8)^bA^	(32.7±0.9)^bB^
T1	(17.05±0.06)^b^	(9.5±0.2)^bc^	(67.52±0.07)^b^	(0.34±0.05)^c^	(76.1±6.2)^b^	(41.9±0.7)^aA^	(26.50±0.05)^dB^
T2	(17.7±0.2)^a^	(9.66±0.09)^b^	(68.0±0.2)^a^	(0.42±0.05)^b^	(85.7±2.4)^a^	(32.55±0.03)^cA^	(26.1±0.5)^dB^
T3	(16.73±0.09)^b^	(10.2±0.1)^a^	(67.55±0.09)^b^	(0.35±0.04)^c^	(59.0±2.4)^c^	(23.8±0.5)^dB^	(27.5±0.6)^cA^
T4	(17.8±0.1)^a^	(9.2±0.2)^c^	(67.8±0.1)^a^	(0.77±0.03)^a^	(64.2±2.5)^b^	(41.10±0.06)^aA^	(37.5±0.4)^aB^

Mean values with the same lowercase/uppercase letter in superscript within a column did not differ significantly (Tukey’s, test/Student’s *t*-test p>0.05), *N*=3. Standard=4% soy protein, T1=0.75% collagen fibre and 3.25% soy protein, T2=1.5% collagen fibre and 2.5% soy protein, T3=0.75% collagen powder and 3.25% soy protein, and T4=1.5% collagen powder and 2.5% soy protein

Regarding lipid content, a variation in the formulations was noted, with values ranging from 8.22% (standard) to 10.2% (T3), given that T3 had statistically higher (p<0.05) lipid content than the other formulations. Variations in lipid content were possibly due to the changes in the raw materials, *i.e.* the fraction of fat removed from the meat.

**Fig. 4:**
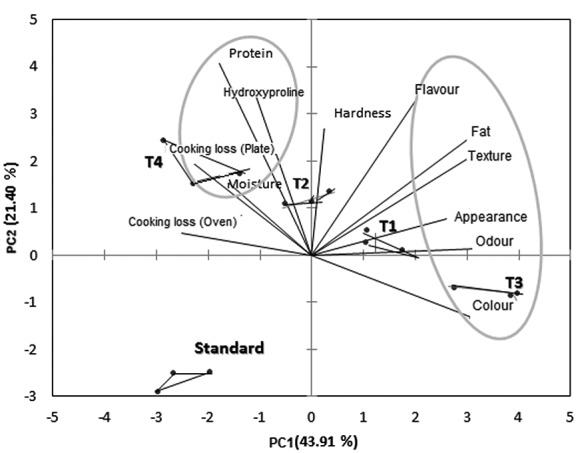
Principal component analysis (PCA) of the hamburger formulations on the first day of storage. Standard=4% soy protein, T1=0.75% collagen fibre and 3.25% soy protein, T2=1.5% collagen fibre and 2.5% soy protein, T3=0.75% collagen powder and 3.25% soy protein, and T4=1.5% collagen powder and 2.5% soy protein

Formulations T2 and T4, containing 1.5% fibre and powdered collagen respectively, were significantly different (p<0.05) from the standard and formulations T1 and T3 (Table 2) and had slightly higher moisture values. Positive correlation was confirmed by the principal component analysis (Fig. 4). Such results are explained by the high water retention capability of the collagen, which reduces water loss during freezing. According to Pietrasik and Janz (*30*), non-meat proteins exhibit similar behaviour to meat proteins, promoting water retention, higher binding, and occupying the interstitial spaces in the gel matrix.

Hydroxyproline content served as a parameter to establish collagen amount in meat and meat products (*3*, *25*). Significant differences (p<0.05) in hydroxyproline mass fraction were observed among the developed formulations (Table 2). All formulations with added collagen showed higher hydroxyproline mass fractions than the standard sample. However, formulations T1 and T3, with 0.75% collagen fibre and powdered collagen respectively, were not significantly different (p<0.05) from each other.

**Fig. 5:**
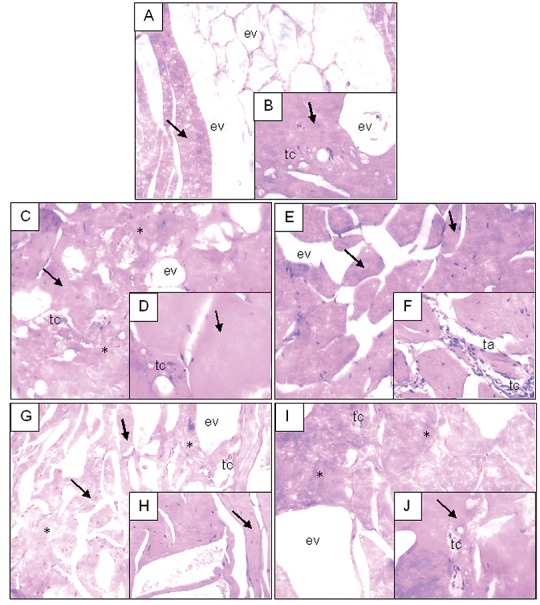
Photomicrographs of hamburger formulations with a detail to the right. Legend: A and B=standard, C and D=formulation T1, E and F=formulation T2, G and H=formulation T3, I and J=formulation T4. Muscle tissue (arrows), adipose tissue (ta), conjunctive tissue (tc), tissue disorganization (*), and empty spaces (ev). Magnification: 10× (A, C, E, G and I) and 25× (B, D, F, H and J)

It was also noted that formulation T4, containing 1.5% powdered collagen, had the highest hydroxyproline content (0.77 g/100 g), 4.8 times higher than the standard formulation. The results found are in accordance with the ones reported by Prestes *et al.* (*29*), where formulations of chicken ham containing a mixture of collagen had higher values of hydroxyproline. Formulation T2 (1.5% collagen fibre) showed increased (p<0.05) hardness (85.7 N) (Table 2 and Fig. 5). When only collagen fibre was added, it resulted in a higher compressive strength and higher shear force in the samples due to the physical structure and larger particle size, which could be related to how high collagen contents in emulsions increase hardness and rigidity, while reducing the mass stability (*31*). In addition, by retaining water chemically through the protein matrix and swelling when in contact with water, collagen fibre alters the texture and cohesion of the hamburger mix, increasing the final product firmness (*32*). This behaviour was also observed by Li (*33*) when adding collagen to the preparation of cooked ham. In that case, collagen caused an increase in hardness from 11.96 to 16.91 N, suggesting that small size proteins affected the texture of the ham.

Mass loss of the hamburger samples prepared in the conventional oven (Table 2) on the first day of storage was on the whole higher than of the ones prepared on the grill (except T3). It must be pointed out that mass loss rate of T3 formulation was lower, which may be better visualized in the multivariate analysis in Fig. 4. Lower mass loss after freeze-thaw and reheating process was found of samples with powdered collagen (Table 2), a phenomenon explained by its greater interaction with the ingredients and additives present in the formulations, creating a cohesive mass. The addition of collagen to meat products as a binder is advantageous; at low levels functional collagen proteins promote an increase in cooking yield due to their gelling and water-binding properties (*34*). According to Pietrasik (*35*), the higher the percentage of added collagen, the lower the release of water due to a greater number of bonds between the polypeptide chains during cooking (formation of a dense protein matrix).

Table 3 shows the results of sensorial evaluation of the hamburger formulations. A significant difference (p<0.05) could be noticed among the formulations. On the whole, formulation T3, made with 0.75% powdered collagen, was the one that received the highest scores (in all attributes) compared to the other formulations. It also had the highest general acceptability of 81% (Table 3).

**Table 3:** Profile of sensorial characteristics of the hamburger formulations on first storage day

Formulation	Sensory attribute					
Appearance	Colour	Odour	Flavour	Texture	General acceptance
Standard	(4.5±0.1)^b^	(4.75±0.2)^b^	(4.7±0.2)^b^	(3.8±0.3)^b^	(3.9±0.2)^c^	(5.4±0.2)^c^
T1	(5.4±0.6)^ab^	(5.25±0.3)^ab^	(5.9±0.2)^a^	(6.2±0.1)^a^	(5.8±0.3)^ab^	(6.6±0.4)^b^
T2	(5.2±0.3)^ab^	(5.08±0.3)^ab^	(5.2±0.3)^ab^	(5.4±0.5)^a^	(5.5±0.3)^ab^	(6.4±0.3)^b^
T3	(6.1±0.1)^a^	(5.92±0.2)^a^	(5.9±0.3)^a^	(6.2±0.3)^a^	(6.3±0.3)^a^	(7.3±0.3)^a^
T4	(5.2±0.3)^ab^	(4.83±0.2)^b^	(4.8±0.2)^b^	(6.2±0.3)^a^	(5.1±0.3)^b^	(6.1±0.3)^b^

Mean values with the same lowercase letters in superscript within a column do not differ significantly at the 95% level (Tukey’s test), *N*=3. Standard=4% soy protein, T1=0.75% collagen fibre and 3.25% soy protein, T2=1.5% collagen fibre and 2.5% soy protein, T3=0.75% collagen powder and 3.25% soy protein, and T4=1.5% collagen powder and 2.5% soy protein

In general, the panellists positively accepted the replacement of soy protein with collagen in hamburgers. It is assumed that such substitutes enhance the acceptability of the hamburgers, as well as help to improve their physical properties, especially in the case of formulation T3. In contrast, the standard formulation obtained the lowest scores. In terms of flavour, it was noted that all formulations containing collagen received higher scores and differed statistically (p<0.05) from the standard sample. These results agree with Sousa *et al.* (*32*), who verified higher texture scores of frankfurter-type sausages containing different collagen mass fractions (25 to 75%) and attributed this effect to the gelatinization property of collagen.

Table S1 and Fig. 4 show, respectively, the Pearson correlation and principal component analysis (PCA) for the physi- cochemical and sensorial variables of the hamburger samples on the first day of storage. The variables are shown as vectors (Fig. 4); the longer the vector, the greater the sample variability. The samples were represented by triangles, where each vertex represented a repetition (*N*=3). It was observed that there was good discrimination among the formulations. The first (PC1) and second (PC2) principal components corresponded to 65.31% of total variance. PC1 contributed with 43.91%, and PC2 contributed with 21.40%.

The values obtained with the Pearson correlation validated the correlation among the parameters observed in the PCA (Fig. 4), with protein presenting a positive correlation (Table S1) with hydroxyproline content (0.666), mass loss during preparation on the grill (0.521) and moisture (0.556). Formulation T4 was the closest to these vectors, also confirmed by the values shown in Table 2, *i.e*. formulation T4 had the highest protein (17.8 g/100 g) and hydroxyproline (0.77 g/100 g) contents. Formulation T3 (0.75% powdered collagen and 3.25% soy protein) received the best sensorial scores (Table 3 and Fig. 4). Positive correlations (Table S1 and Fig. 4) were also verified between flavour and fat content, and between texture (>0.70) and fat, appearance, colour, odour and flavour. However, with increased hardness (instrumental texture), there was a decrease in oven mass loss.

Due to its properties, such as extender, emulsifier, texture improver, and its nutritional value, collagen has great application potential in the industry of restructured and emulsified meat products, providing better technological performance and economic results. Collagen beneficially participates in meat emulsions in the range from 15 to 18%, mainly aiding the texture and stability of the mass (*36*), reducing water loss in defrosting and cooking.

### Histological characteristics of hamburger formulations

Fig. 5 shows the photomicrographs of hamburger formulations. The use of histological methods allowed for the qualitative analysis of muscular, adipose and conjunctive tissues. The standard sample (A and B in Fig. 5) shows muscular tissue (arrows) associated with dense conjunctive tissue (tc). Empty spaces (ev) can be noticed between the muscular tissues and/or spaces with adipose tissue. It achieved a cellular organization classified as “good”, but without consistency. It was the sample with the worst texture according to sensorial evaluation (Table 3 and Fig. 4). Formulation T1 (C and D in Fig. 5) shows tissue disorganization (*), *i.e.* empty spaces (ev) dispersed among muscular cells (arrows), possibly adipose tissue, as well as dense conjunctive tissue (tc). The bad cellular organization and added collagen fibre caused an increase in the hamburger hardness (Table 2). Formulation T2 (E and F in Fig. 5) has a muscular cell organization with peripheral nuclei (arrows) and some adipose cells (ta). It shows a non-emulsified conjunctive tissue with spaces between the conjunctive tissues, with cellular organization. The non-emulsion of the connective tissue caused the highest values of hardness due to the higher mass fraction of collagen fibre (1.5%). Formulation T3 (G and H in Fig. 5) showed intense tissue disorganization (*) since there was not any difference among muscle (arrows), conjunctive and adipose tissues, technologically qualified as emulsified material, providing to the better hamburger texture (Table 2) and sensory (Table 3) characteristics. Formulation T4 (I and J in Fig. 5) showed intense tissue cohesion, but due to little difference among adipose, muscle and conjunctive (*) tissues, it also had greater mass loss during heat treatment (Table 2 and Fig. 4).

## CONCLUSIONS

Powdered collagen and collagen fibre are products with distinctive characteristics, mainly in terms of their protein composition, hydroxyproline content and gel strength. The fibre and powdered collagen have a molecular mass of 50 to 100 and 100 kDa respectively, and higher protein content (97.81 and 96.87 g/100 g) and gel strength (146 315 and 91 888 Pa) respectively than the standard sample. Powdered collagen is more amorphous than the fibre one. In the hamburger formulations with 1.5% collagen, there was an increase in protein and moisture content. Sensorial analysis showed that the hamburger formulation containing 0.75% powdered collagen received the best colour, appearance, texture and general acceptance evaluation. The histological analysis of the same formulation showed intense tissue disorganization, typical for emulsified material, with adipose tissue mixed with conjunctive one. The mass loss by baking in oven diverged among the hamburger formulations, but it was higher than when using the grill. This knowledge is useful for the development of novel strategies in which the mass fractions and collagen preparations are optimized to promote specific and desired technological attributes for healthier meat products. In addition, the use of bovine collagen (fibre and powdered) in hamburger can be an alternative to increase the intake of collagen by the consumer, contributing to the prevention of joint diseases and generate an opportunity for the industry to produce new functional meat products.
